# Transgene-free mouse embryo models from chemical reprogramming reach early organogenesis

**DOI:** 10.1186/s13619-025-00259-5

**Published:** 2025-09-04

**Authors:** Xiu Yu, Jichang Wang

**Affiliations:** 1 Key Laboratory for Stem Cells and Tissue Engineering (Sun Yat-Sen University), Ministry of Education, Guangzhou, 510080 China; 2 Department of Histology and Embryology, Zhongshan School of Medicine, Sun Yat-Sen University, Guangzhou, 510080 China; 3 Reproductive Medicine Center, The First Affiliated Hospital, Sun Yat-Sen University, Guangzhou, 510080 China

## Abstract

Embryo models derived from pluripotent stem cells (PSCs) have become powerful tools for dissecting mammalian embryonic development and advancing regenerative medicine. Two recent studies in *Cell* and *Cell Stem Cell* report major advances in generating mouse embryo models that replicate development up to early organogenesis (equivalent to embryonic day 8.5~8.75). Li et al. describe a purely chemical strategy to reprogram mouse embryonic stem cells (mESCs) into induced embryo founder cells (iEFCs) capable of forming complete embryo models (iEFC-EMs). In parallel, Yilmaz et al. demonstrate transgene-free generation of post-gastrulation models (TF-SEMs) from naive mESCs and induced pluripotent stem cells (iPSCs) using a similar chemical cocktail. Both models faithfully recapitulate key developmental events, including gastrulation, neural tube formation, cardiogenesis, and somitogenesis. These advances not only deepen understanding of early mammalian development but also pave the way for applications in regenerative medicine and disease modeling.

## Main text

Understanding mammalian embryogenesis is a fundamental challenge in developmental biology. Ethical and technical restrictions on studies of early human embryos have long limited insights into this intricate process. In recent years, stem cell-based embryology has emerged as a powerful approach to reconstruct early developmental events in vitro. Over the past decade, notable advances have been made in generating embryo-like structures from PSCs. For instance, PSCs cultured under defined conditions supplemented with key morphogens (e.g., BMP4, Wnt agonists, and Nodal inhibitors) can self-organize into gastruloids that partially mimic primitive streak formation and germ layer specification (Beccari et al. 2018; Xu et al. 2021). Yet, these models lack extra-embryonic tissue contributions, which are essential for normal development, and often arrest at early gastrulation-like stages.

To address these limitations, researchers have developed co-culture systems combining ESCs, trophoblast stem cells (TSCs), and extra-embryonic endoderm (XEN) cells to generate self-assembling embryo-like structures. These synthetic models can replicate key developmental milestones including epiblast differentiation, lumenogenesis, and egg cylinder morphogenesis. Rotating culture platforms have extended their development to stages equivalent to E8.5 in natural embryos. However, variability in starting cell populations, reliance on transgenic lineage induction, and incomplete functional equivalence of TSC/XEN cells continue to limit reproducibility and developmental fidelity of synthetic embryo models (Amadei et al. 2022; Tarazi et al. 2022).

Recently, Li and colleagues (Li et al. n.d.) addressed these issues with a chemical-based strategy, converting mESCs into embryo founder cells (iEFCs) resembling the 8~16 cell stage of early mouse development. The iEFCs co-express the pluripotency marker OCT4 with lineage specifiers CDX2 (trophectoderm) and GATA6 (primitive endoderm), retaining competence for both embryonic and extra-embryonic fates. Without transgenes or cell-types mixing, iEFCs self-assemble into blastocyst-like structures, containing TE-like, PrE-like, and epiblast-like compartments. On further optimized culture, iEFC-derived embryo models (iEFC-EMs) can progress through gastrulation to early organogenesis with 35% efficiency—mimicking natural embryos up to E8.75—forming forebrain/midbrain/hindbrain regions, a beating heart tube, 6~14 pairs of somites, and migrating primordial germ cells. The iEFC-EMs model overcomes developmental limitations of blastoids that cannot progress to early organogenesis and of gastruloids that lack extra-embryonic tissue contributions. The ability to generate all lineages from solely starting cell type (ESCs) through small molecule induction is a significant improvement over co-culture-based methods, as it simplifies the experimental setup and reduces variability.

In parallel, Yilmaz and colleagues (Yilmaz et al. n.d.) developed transgene-free embryo models (TF-SEMs) from naïve mESCs and induced pluripotent stem cells (iPSCs) using a chemical cocktail closely resembling Li’s, with minor modifications. While TF-SEMs advanced to stages equivalent to E8.5~E8.75, their developmental efficiency was lower: 34.36% formed egg cylinders compared to 65% for iEFC-EMs, and only 3.31% reached early organogenesis versus 35.2% for iEFC-EMs. These differences likely reflect intrinsic cell line properties, variation in extra-embryonic cell induction, and/or protocol-specific limitations.

Both studies demonstrate that precisely tuned retinoic acid (RA), Wnt, and TGF-β signaling can direct mouse naïve PSCs to generate all blastocyst lineages, including TE and PrE—contradicting the longstanding dogma that the mouse naive PSCs cannot be readily converted into the TE lineage. The high developmental potential of these embryo models appears to stem from the rapid emergence of heterogeneous populations co-expressing EPI, TE, and PrE markers, with high-fidelity PrE specification proving essential for proper gastrulation (Linneberg-Agerholm et al. 2024). In iEFCs, more than 80% of cells co-express Oct4, Cdx2, and Gata6, recapitulate the transcriptional profile of early embryos (8 to 16-cell stage) and exhibiting chromatin accessibility at promoter/enhancers of key lineage specifiers (e.g., Cdx2, Gata6). These unique transcriptomic and epigenetic features underpin their ability to differentiate into EPI, TE, and PrE lineages. Yilmaz et al. similarly detected a “mixed-identity” subpopulation in mouse PSCs under certain chemical condition. Prior work has shown that WNT and RA activation promotes PrE induction, while TGF-β1 inhibition enhances hypoblast specification in humans (Ohinata et al. 2022; Okubo et al. 2024). Notably, simultaneous WNT/RA activation can also induce a 2-cell-like totipotent state from PSCs (Iturbide et al. 2021; Xu et al. 2022). However, Yilmaz et al. used cell trajectory analysis to show that PrE and TE-like cells arise directly from PSCs without passing through 2CLC intermediates. This finding aligns with our independent single-cell transcriptomic analysis of CHIR/RA-induced totipotent-like stem cells (data not shown), which likewise identified minor PrE/TE-like populations emerging without a 2CLC transition. Despite these insights, the precise regulatory mechanisms by which WNT and RA signaling orchestrate extra-embryonic lineage specification remains to be determined. Addressing these questions will be crucial for refining embryo models and advancing our understanding of early mammalian development.

In addition, the ability of iEFCs to generate all blastocyst lineages marks a pivotal milestone in embryonic development. To direct this lineage commitment, Li et al. employed a temporally controlled cocktail comprising the LATS inhibitor TRULI (promoting TE specification), Activin A, XAV939 (WNT inhibition), BMP4, and FGF4 (supporting TE formation). Within 36 h, OCT4/CDX2/GATA6 triple-positive cells resolve into PrE-, TE-, and EPI-like subpopulations with transcriptomic profiles closely matching their E4.5 in vivo counterparts. Compared with ESC/TSC/XEN co-cultures or transgenic approaches, iEFC models better preserve preimplantation-like epigenetic states, complete extra-embryonic potential, and physiologically relevant cell–cell interactions.

Although these embryo models closely resemble in vivo embryos at E8.5~E8.75 in overall morphology and multiple cell types, it needs to investigate whether their three-dimensional architecture, lineage balance, and intercellular interactions faithfully recapitulate endogenous developmental processes. Reported abnormalities include cardiac hypertrophy and neural tube defects in TF-SEMs, as well as reduced trophoblast function in iEFC-EMs. Moreover, cell line–specific variability influences both developmental efficiency and lineage fidelity. Notably, despite their robust in vitro developmental potential, Yilmaz et al. reported that 4-day cultured aggregates exhibited low implantation efficiency upon transfer into mouse uteri and failed to form normal embryonic structures. Elucidating the regulatory mechanisms underlying this discrepancy between in vivo and in vitro developmental potential will be key to optimizing culture condition and promoting embryo models towards more advanced developmental states.

Future work should (1) optimize media and culture to mimic uterine microenvironment to promote these embryo models development into more mature stage, (2) correct developmental defects in the heart and neural tube via signaling modulation (e.g., optimizing BMP and FGF concentrations), (3) standardize the initial state of cells (e.g., epigenetic modifications of naive PSCs) to reduce variability, (4) employ automated screening for high-quality models, and (5) extend chemical-based strategies to establish embryo models in humans and other species.

## Conclusion

These groundbreaking studies establish efficient, transgene-free mouse embryo models, overcoming limitations of prior systems and enabling complete embryo formation from solely stem cell types. They provide powerful platforms for investigating early development, modelling congenital defects, and assessing embryotoxicity. Extending their developmental window and functional completeness will be critical for translating these models into broader basic and biomedical applications.

## Data Availability

Not applicable.
